# Resveratrol and its Related Polyphenols Contribute to the Maintenance of Genome Stability

**DOI:** 10.1038/s41598-020-62292-5

**Published:** 2020-03-25

**Authors:** Yusuke Matsuno, Yuko Atsumi, Md. Alauddin, Md. Masud Rana, Haruka Fujimori, Mai Hyodo, Atsuhiro Shimizu, Tomoki Ikuta, Hiroko Tani, Hidetaka Torigoe, Yoshimichi Nakatsu, Teruhisa Tsuzuki, Michio Komai, Hitoshi Shirakawa, Ken-ichi Yoshioka

**Affiliations:** 1 Division of Carcinogenesis and Cancer Prevention, National Cancer Center Research Institute, 5-1-1 Tsukiji, Chuo-ku Tokyo, 104-0045 Japan; 2 Department of Applied Chemistry, Faculty of Science, Tokyo University of Science, 1-3 Kagurazaka, Shinjuku-ku Tokyo, 162-8601 Japan; 3 Laboratory of Nutrition, Graduate School of Agricultural Science, Tohoku University, 468-1, Aramaki Aza Aoba, Aoba-ku Sendai, 980-8572 Japan; 4 Biological Science and Technology, Tokyo University of Science, 6-1-1 Niijuku, Katsushika-ku Tokyo, 125-8585 Japan; 5 Institute for Bee Products and Health Science, Yamada Bee Company, Inc., 194 Ichiba, Kagamino-cho, Tomata-gun Okayama, 708-0393 Japan; 6 Department of Medical Biophysics and Radiation Biology, Faculty of Medical Sciences, Kyushu University, Maidashi, Higashi-ku Fukuoka, 812-8582 Japan; 7 International Education and Research Center for Food and Agricultural Immunology, Graduate School of Agricultural Science, Tohoku University, 468-1 Aramaki Aza Aoba, Aoba-ku Sendai, 980-8572 Japan

**Keywords:** DNA damage and repair, Genomic instability, Molecular biology, Natural products, Cell biology, Senescence, Cancer prevention

## Abstract

Genomic destabilisation is associated with the induction of mutations, including those in cancer-driver genes, and subsequent clonal evolution of cells with abrogated defence systems. Such mutations are not induced when genome stability is maintained; however, the mechanisms involved in genome stability maintenance remain elusive. Here, resveratrol (and related polyphenols) is shown to enhance genome stability in mouse embryonic fibroblasts, ultimately protecting the cells against the induction of mutations in the ARF/p53 pathway. Replication stress-associated DNA double-strand breaks (DSBs) that accumulated with genomic destabilisation were effectively reduced by resveratrol treatment. In addition, resveratrol transiently stabilised the expression of histone H2AX, which is involved in DSB repair. Similar effects on the maintenance of genome stability were observed for related polyphenols. Accordingly, we propose that polyphenol consumption can contribute to the suppression of cancers that develop with genomic instability, as well as lifespan extension.

## Introduction

Most cancers are associated with genomic instability, which can be categorised as chromosomal instability or microsatellite instability (MSI)^1^. Genomic destabilisation is a major cause of mutations, including those in cancer-driver genes, and can lead to clonal evolution of cells with abrogated defence systems, such as those containing mutations in the ARF/p53 pathway^2^. Genome stability maintenance would likely prevent the formation of mutations and suppress cancer development; however, it is still unclear if genome stability can be maintained *in vivo* and whether this process can indeed suppress the occurrence of cancer.

Genomic instability is caused by the erroneous repair of DNA double-strand breaks (DSBs); paradoxically, the DNA repair systems of most cancers that develop with genomic instability are genetically normal^3^. The mechanisms by which normal cells accumulate DSBs remain unclear, but DSBs widely accumulate in pre-cancerous cells and are accompanied by genomic instability^2,4–6^. *In vitro*, replication stress-associated DSBs and the associated genomic instability are observed in cells subjected to aberrant growth stimulation^2^ or overexpression of oncogenes such as c-Myc and E2F1^6–8^.

Possibly reflecting the correlation between cancer development and age, DSBs accumulate with age *in vivo* and with cultivating passages *in vitro*^9^, suggesting that ageing cells are defective in DSB repair. DSB repair deficiency is probably related at least in part to a reduction in the level of H2AX. This histone mediates DSB repair and is required for genome stability maintenance, and H2AX expression levels are attenuated when the growth rate of normal cells slows down^10,11^. In fact, such cells are defective in repairing replication stress-associated DSBs^10^, although they are still able to repair DSBs caused by γ-rays because H2AX is transiently stabilised under these conditions^12^.

A number of animal studies have shown that regular polyphenol consumption can contribute to cancer suppression in association with lifespan extension^13–18^. Such cancer-suppressive effects of polyphenols have been reported for a wide variety of cancers that generally arise with genomic instability at advanced ages, such as skin^19^, prostate^20^, colon^21^ and breast cancers^22^. It is possible that the anti-cancer and anti-aging effects of polyphenols are related to their positive effects on genome integrity.

In this study, we examined the effects of polyphenols on genome stability maintenance, DSB repair and genomic instability-associated cancer suppression. We found that resveratrol, a polyphenol found in red wine, and related polyphenols maintain genome stability by inducing DSB repair, thereby contributing to the suppression of cancer that develops with genomic instability.

## Results

### Resveratrol contributes to genome stability

**Figure 1:**
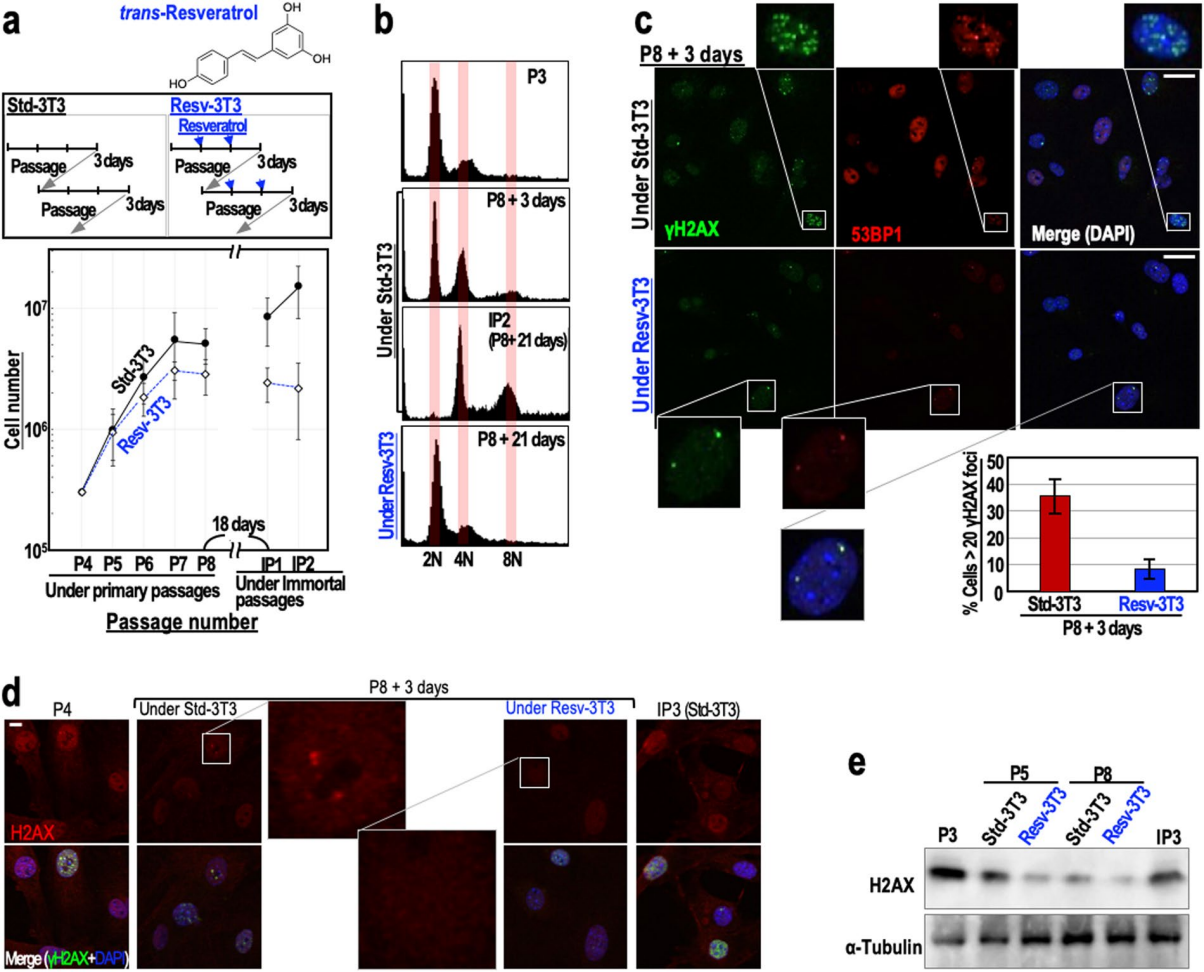
Regular resveratrol treatment maintains genome stability and suppresses DSB accumulation. (**a**) The immortalisation of MEFs cultivated under the Std-3T3 or Resv-3T3 protocol as indicated. In the Resv-3T3 protocol, the cells were treated with 2.5 μM resveratrol regularly. In the lower graph, data are represented as the mean ± s.d. (n = 3 biologically independent experiments). (**b**) The effects of cultivation of MEFs under the Std-3T3 or Resv-3T3 protocol on chromosomal instability (tetraploidy). (**c**) Statuses of the γH2AX and 53BP1 foci in MEFs at a growth-arrested stage (P8 + 3 days) following cultivation under the Std-3T3 or Resv-3T3 protocol. The numbers of γH2AX foci were quantified and data are represented as the mean ± s.d. (n = 3 biologically independent experiments). (**d**,**e**) Immunostaining (**d**) and immunoblot (**e**) analyses of H2AX in quiescent MEFs grown under the Std-3T3 or Resv-3T3 protocol. (**c**,**d**) Scale bars, 10 μm.

To examine the effects of resveratrol on genome stability, we monitored the immortalisation of mouse embryonic fibroblasts (MEFs) (Fig. 1a). When cultivated under the ‘3T3 protocol’, MEFs initially show serial proliferation, but then undergo growth-arrested senescence, and subsequently immortalise with genomic instability (tetraploidy)^8,10^ and mutations in the ARF/p53 pathway^11,23^. As expected, MEFs grown under the standard 3T3 protocol (Std-3T3) immortalised with tetraploidy, but MEFs that were regularly treated with 2.5 μM resveratrol (Resv-3T3 protocol) displayed continuous genome stability and were protected against immortalisation (Fig. 1a,b). Supporting these results, the percentages of cells with two nuclei or micronuclei, the levels of which increase during genomic destabilisation, were lower for the Resv-3T3 group than for the Std-3T3 group (Supplementary Fig. S1a). The induction of aberrant nuclei by γ-ray irradiation was also reduced in the presence of resveratrol, although the differences between the control cells and resveratrol-treated cells were not statistically significant (Supplementary Fig. S1b). These results indicate that continuous resveratrol treatment contributes to genome stability maintenance. Given that MEFs generally immortalise with abrogation of the ARF/p53-dependent barrier^11,23^, resveratrol may suppress the induction of mutations in cancer-driver genes via the maintenance of genome stability.

Genomic destabilisation (tetraploidisation) in MEFs is generally triggered by replication stress-associated DSBs that accumulate under the Std-3T3 protocol, in association with cellular stress caused by continuous growth acceleration. Since our initial experiment suggested that regular resveratrol treatment can suppress genomic destabilisation, we examined the accumulation of DSBs in passage 8 (P8) MEFs grown under the Std-3T3 and Resv-3T3 protocols, by counting the numbers of γH2AX/53BP1 foci. As expected, the number of γH2AX/53BP1 foci in MEFs grown under the Resv-3T3 protocol was markedly smaller than that in MEFs grown under the Std-3T3 protocol (Fig. 1c). This finding is consistent with the observed genome stabilising effect of resveratrol described above, and suggests that regular resveratrol treatment is associated with the reduction of DSBs and the neutralisation of replication stress.

Normal cells generally undergo growth arrest after serial proliferation. At this stage, two different cellular states can be discriminated: (1) a senescent state associated with γH2AX foci accumulation and an increased risk of genomic destabilisation, and (2) a quiescent state associated with down-regulated H2AX expression and maintenance of genome stability^24^. Therefore, we compared the expression levels of H2AX in MEFs grown under the Std-3T3 and Resv-3T3 protocols. As expected, an immunostaining analysis revealed that the H2AX signal was substantially lower in the Resv-3T3 cells than in the Std-3T3 cells at the growth-arrested stage (P8 + 3 days) (Fig. 1d). A western blotting analysis confirmed that, as seen in quiescent cells, H2AX expression was down-regulated in both cell groups at the growth-arrested stage, but the reduction was larger for the resveratrol-treated cells than for those grown under the standard protocol (Fig. 1e). Together with the observed reduction in γH2AX/53BP1 foci formation following resveratrol treatment, these findings indicate that resveratrol contributes to DSB reduction and genome stability maintenance by inducing a quiescent state with down-regulated H2AX levels.

### Resveratrol enables DSB repair through transient induction of H2AX

**Figure 2:**
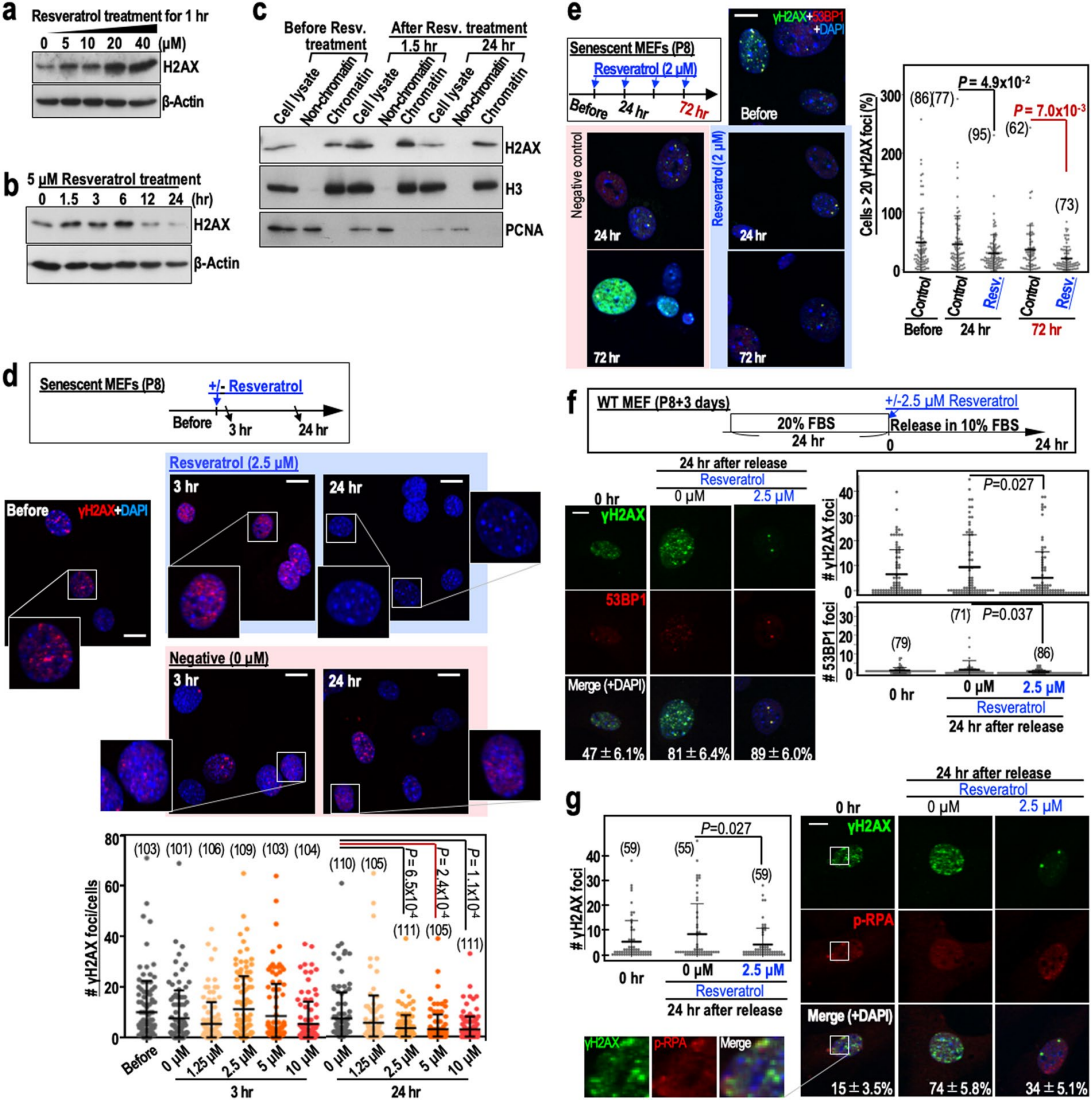
Resveratrol treatment of MEFs transiently stabilises H2AX, leading to reduced numbers of γH2AX foci. (**a**) H2AX expression in senescent MEFs (passage 8) treated with different concentrations of resveratrol. The dose-dependent effects of resveratrol were assessed 1 hr after treatment. (**b**) The time-dependent effects of 5 μM resveratrol on H2AX expression in senescent MEFs (passage 8). (**c**) H2AX expression in senescent MEFs (passage 8) treated with resveratrol (5 μM) and then fractionated into the chromatin and non-chromatin fractions before and 1.5 and 24 hr after treatment. Histone H3 (H3) and PCNA were used as controls. (**d**) Immunofluorescence analyses examining the effect of resveratrol (2.5 μM) on the number of γH2AX foci in senescent MEFs (n numbers are indicated in the graph). The dose-dependent effects of resveratrol were assessed 3 and 24 hr after treatment. (**e**) The effects of multiple resveratrol treatments (2 μM) on the number of γH2AX foci in senescent MEFs. Reductions in the number of γH2AX foci were assessed 24 hr (single treatment) and 72 hr (three treatments) after resveratrol exposure. Data are represented as the mean ± s.d. (n = 3 biologically independent experiments). (**f**,**g**) The effects of resveratrol (2.5 μM) on the numbers of merged γH2AX/53BP1 (**f**) and γH2AX/p-RPA (**g**) foci in senescent MEFs. The MEFs were treated as shown in the workflow (**f**). γH2AX, 53BP1 and p-RPA were detected by immunofluorescence (n numbers are indicated in the graph). The percentages of the γH2AX foci that merged with 53BP1 or p-RPA foci (mean ± s.e.) are indicated in each image. Data in the graphs are represented as the mean ± s.d. Scale bars, 10 μm. *P*-values were calculated by two-tailed Welch’s *t*-tests.

After P8, MEFs are at an increased risk of genomic destabilisation due to a deficiency in the repair of DSBs caused by the replication stress that arises during cultivation under Std-3T3 conditions. Although it is unclear how normal cells become defective in DSB repair after serial proliferation, the process is thought to involve a reduction in H2AX levels^10,11^. It is possible that the repair of DSBs in such cells occurs via transient up-regulation of H2AX, as occurs after γ-ray irradiation of cells^12^. To test this hypothesis, we performed a 24 hr time-course analysis of H2AX levels in MEFs treated with 5 µM resveratrol (Fig. 2a,b). As expected, H2AX was transiently induced after resveratrol treatment of P8 MEFs, with the peak induction at 6 hr post-treatment. In addition, H2AX transiently induced by resveratrol was efficiently incorporated into chromatin but its level decayed within 24 hr (Fig. 2c).

To examine its effect on DSB repair, we treated P8 MEFs with different concentrations of resveratrol and monitored γH2AX foci status. As expected, the number of γH2AX foci was reduced significantly 24 hr after treatment of MEFs with 2.5 µM resveratrol (Fig. 2d). In addition, the number of γH2AX foci was reduced further by multiple resveratrol treatments (Fig. 2e). The γH2AX foci largely merged with 53BP1 foci, which accumulate at DSB sites (Fig. 2f), and with phosphorylated RPA32 (p-RPA at Ser33) foci, which arise in association with replication stress (Fig. 2g). The numbers of these foci were reduced in the presence of resveratrol (Fig. 2f), suggesting efficient repair of DSBs in the presence of resveratrol.

Resveratrol may modulate multiple biological pathways and may induce apoptosis at high doses^25^. To compare the conditions that lead to DSB repair and apoptosis, the effects of three concentrations of resveratrol (2.5, 25 and 250 μM) on multiple cell types were examined after pre-treatment of cells with 0.25 mM hydroxyurea to weakly induce replication stress. As expected, the level of cleaved caspase-3, a marker of apoptosis induction, was increased after treatment of MEFs (P4), HUC-F2 cells (P9) and WI38 cells (P6) with 250 μM resveratrol, and after treatment of HeLa cells with 25 μM resveratrol (Supplementary Fig. S2a). Under these conditions, the numbers of γH2AX foci were not increased prior to the induction of apoptosis (Supplementary Fig. S2b; see 25 μM for HeLa and 250 μM for MEFs), implying the separation of apoptosis induction from damage responses. Notably, these findings indicate that the concentration of resveratrol that leads to DSB reduction is much lower than that required for the induction of apoptosis.

Normal cells treated with hydroxyurea usually undergo growth arrest and hence show only a limited response to damage^26^. Therefore, we also examined the effect of resveratrol on the numbers of replication stress-associated DSBs 24 hr after γ-ray irradiation (2 Gy) of MEFs, HeLa cells and WI38 cells (Supplementary Fig. S3a), because replication stress-associated DSBs accumulate after the repair of DSBs that are directly caused by γ-ray exposure^27^. As expected, the number of γH2AX foci that merged with p-RPA (Supplementary Fig. 3b) and 53BP1 was reduced significantly in the presence of 2.5 μM resveratrol (Supplementary Fig. S3a). Furthermore, the numbers of γH2AX foci and 53BP1 foci in MEFs that were γ-ray irradiated with 1 or 5 Gy were also reduced significantly in the presence of 2.5 μM resveratrol (Supplementary Fig. S3c). Based on these findings, we concluded that replication stress-associated DSBs are reduced by treatment of cells with relatively low concentrations of resveratrol.

### Resveratrol-related polyphenols contribute to genome stability

**Figure 3:**
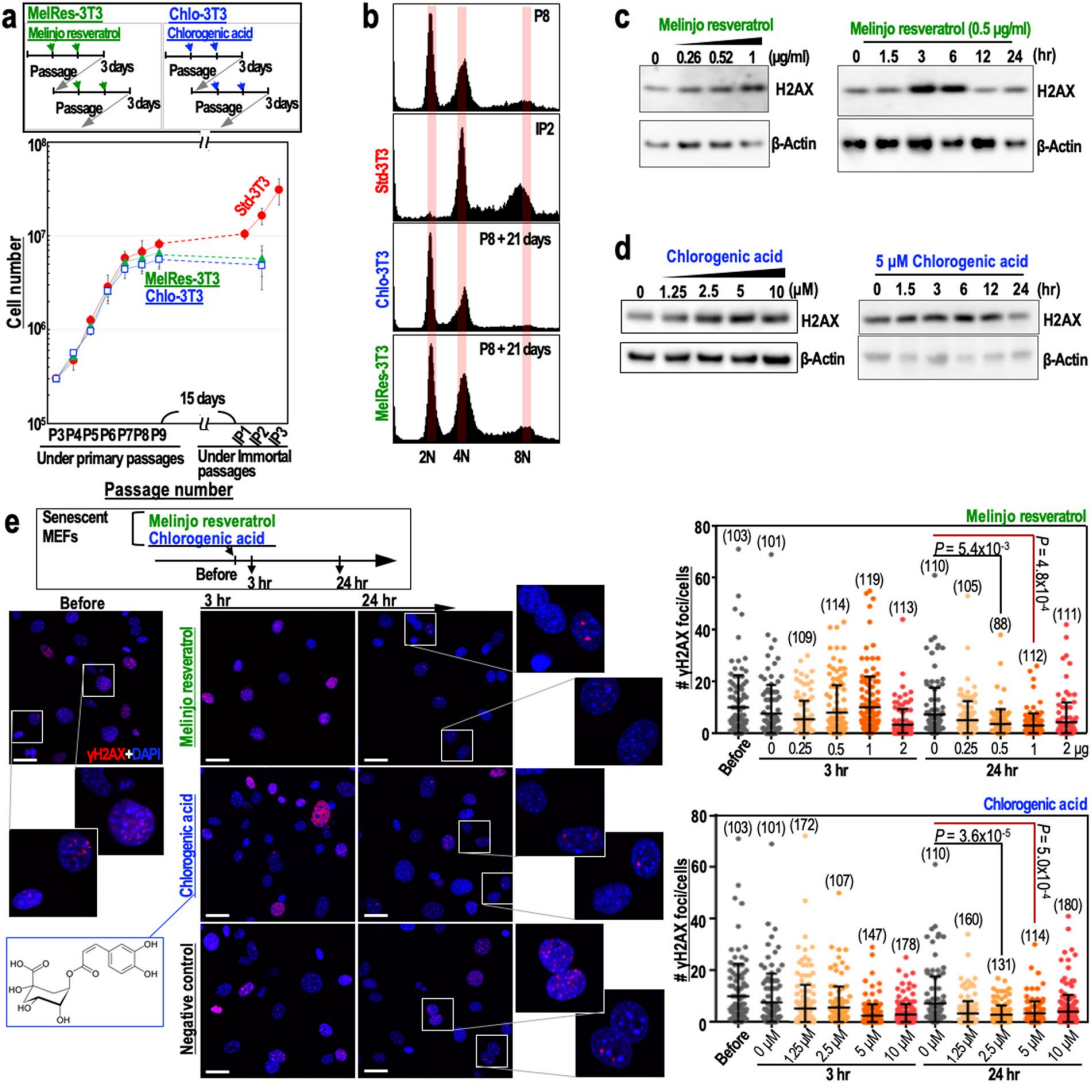
Melinjo resveratrol and chlorogenic acid contribute to genome stability maintenance. (**a**) The immortalisation of MEFs cultivated under the Std-3T3, MelRes-3T3 (melinjo resveratrol) or Chlo-3T3 (chlorogenic acid) protocol as indicated. In the MelRes-3T3 and Chlo-3T3 protocols, MEFs were regularly treated with 0.5 μg/ml melinjo resveratrol and 2.5 μM chlorogenic acid, respectively. In the lower graph, data are represented as the mean ± s.d. (n = 3 biologically independent experiments). (**b**) The effects of cultivation of MEFs under the Std-3T3, MelRes-3T3 or Chlo-3T3 protocol on chromosomal instability (tetraploidy). (**c**,**d**) Time- and dose-dependent effects of melinjo resveratrol (**c**) and chlorogenic acid (**d**) on H2AX expression in senescent MEFs (passage 8). The dose-dependent effects were assessed 3 hr after treatment. The time-dependent effects were determined using 0.5 μg/ml melinjo resveratrol (**c**) or 5 μM chlorogenic acid (**d**). (**e**) The effects of melinjo resveratrol (0.5 μg/ml) and chlorogenic acid (5 μM) on the number of γH2AX foci in senescent MEFs at 3 and 24 hr post-treatment. γH2AX foci were detected by immunofluorescence (n numbers are indicated in the graphs). Data in the graphs are represented as the mean ± s.d. Scale bars, 10 μm. *P*-values were calculated by two-tailed Welch’s *t*-tests.

A number of studies have reported potential benefits of polyphenol consumption on cancer suppression and longevity^28–30^. Therefore, we examined the genome stabilising effects of chlorogenic acid, a polyphenol found in coffee, and melinjo resveratrol, a mixture of multiple resveratrol-associated polyphenols produced in melinjo seeds. Similar to resveratrol (Fig. 1), chlorogenic acid (2.5 μM) and melinjo resveratrol (0.5 μg/ml) inhibited the immortalisation of MEFs (Fig. 3a) and promoted genome stability (Fig. 3b). In addition, as seen for resveratrol, chlorogenic acid and melinjo resveratrol caused a transient induction of H2AX (Fig. 3c,d) and a reduction in the number of γH2AX foci (Fig. 3e). These findings suggest that the broad health benefits of these polyphenols could be due to the maintenance of genome stability, which primarily occurs via the reduction of DSBs.

**Figure 4:**
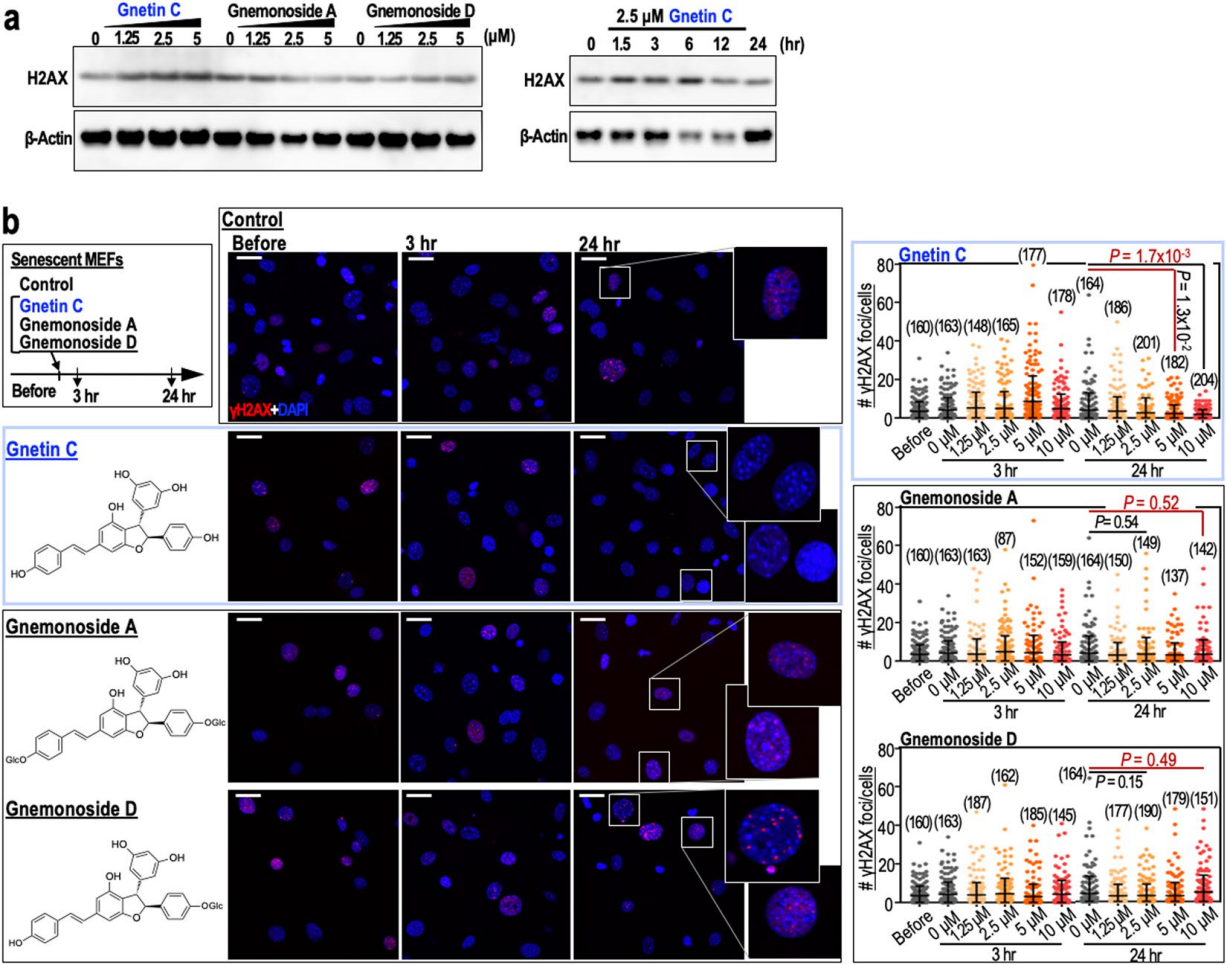
Gnetin C mediates transient stabilisation of H2AX and a reduction in the number of γH2AX foci. (**a**) Dose-dependent effects of gnetin C, gnemonoside A and gnemonoside D, and time-dependent effects of gnetin C on H2AX expression in senescent MEFs (passage 8). The dose-dependent effects were assessed 3 hr after treatment. The time-dependent effect was determined using 2.5 μM gnetin C. (**b**) The effects of gnetin C (2.5 μM), gnemonoside A (2.5 μM) and gnemonoside D (2.5 μM) on the numbers of γH2AX foci in senescent MEFs at 3 and 24 hr post-treatment. Data in the graphs are represented as the mean ± s.d. Scale bars, 10 μm. *P*-values were calculated by two-tailed Welch’s *t*-tests.

Melinjo resveratrol is a mixture of multiple polyphenols, the major components being gnetin C (resveratrol dimer) and its glycosides gnemonoside A and gnemonoside D^31^. To determine which of these components is responsible for the effect of melinjo resveratrol on genome stability, we examined their effects on transient H2AX induction and resulting DSB repair (γH2AX foci reduction) in MEFs. Gnetin C induced transient H2AX induction and DSB repair in P8 MEFs, whereas gnemonoside A and gnemonoside D did not (Fig. 4a,b), indicating that the gnetin C component of melinjo resveratrol contributes to the reduction of DSBs in association with transient H2AX induction.

### Consumption of melinjo resveratrol suppresses cancer development

**Figure 5:**
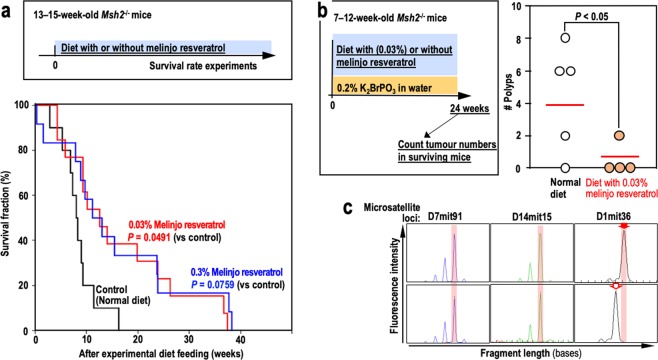
Melinjo resveratrol consumption contributes to the suppression of cancer associated with microsatellite instability. (**a**) Kaplan-Meier curves showing the survival rates of *Msh2*^−/−^ mice fed a normal diet or a diet supplemented with melinjo resveratrol (0.3% or 0.03%). Data were analysed by a Log-Rank test followed by a Holm-Sidak test; n = 10 (control group), n = 13 (0.03% melinjo resveratrol group), n = 12 (0.3% melinjo resveratrol group). (**b**) The numbers of tumours in the small intestines of K_2_BrPO_3_-treated mice fed a normal diet or a diet supplemented with 0.03% melinjo resveratrol. Statistical analysis was performed via a Student’s *t*-test. (**c**) The MSI statuses at three microsatellite loci in tissue from normal organs (top row) and polyps (bottom row). Red arrows indicate the shifted fragment peaks, i.e., MSI.

Based on our results demonstrating the genome stabilising effect of melinjo resveratrol, we used *Msh2*^*−/−*^ mice to examine its effect on cancer suppression and its possible association with lifespan extension. *Msh2*^*−/−*^ mice have high mutation rates^32,33^ that predispose them to cancer (mainly lymphoma) in association with MSI^34^, which is related to the clonal evolution of cells containing mutations in cancer-driver genes^2^. Since genome stability effects of melinjo resveratrol were shown with almost same range with those of resveratrol in weight concentration (Figs. 2d and 3e), melinjo resveratrol were treated with same dose range with previous resveratrol studies shown cancer suppression effects^35,36^. Kaplan-Meir survival curves revealed that *Msh2*^*−/−*^ mice fed a diet containing melinjo resveratrol had a significantly longer lifespan than those fed the control diet (Fig. 5a), indicating that melinjo resveratrol consumption contributes to lifespan extension even in a mismatch repair (MMR)-deficient background.

To evaluate the effect of melinjo resveratrol on cancer incidence, we analysed the sensitivity of *Msh2*^*−/−*^ mice to K_2_BrPO_3_, which primarily induces DNA damage and promotes the formation of MSI-associated cancers in the small intestine^37^. As expected, tumour numbers were significantly lower in mice consuming the 0.03% melinjo resveratrol diet than in those consuming the control diet (Fig. 5b), in which tumours developed with MSI (Fig. 5c). These results support the notion that genomic destabilisation-associated cancers can be suppressed by the consumption of polyphenols that reduce DSBs and maintain genome stability.

## Discussion

The results presented here demonstrate that resveratrol and related polyphenols contribute to genome stability maintenance, primarily via suppression of the DSB level. In an *in vivo* study, the beneficial effects of resveratrol on genome integrity were also associated with the suppression of MSI-associated cancer and a subsequent extension of the lifespan of mice. These results are consistent with those of previous studies reporting suppression of cancers that develop with genomic instability by polyphenol consumption^19–22^.

Cancer generally develops with mutations in cancer-driver genes. Whether the formation of cancer-driver mutations and the resulting disease is avoidable is still a matter of debate^38–40^. The conventional view is that most mutations, including cancer-driver mutations, are randomly induced during DNA replication; therefore, the majority of cancers are unavoidable^41^. However, a recent study reported that, unlike mutations induced during canonical replication, those caused by replication stress along with genomic destabilisation can be avoided through genome stability maintenance^42^. Mutations induced during canonical replication are limited, even in MMR-deficient cells that cannot repair errors caused by replication forks; however, massive numbers of mutations occur under replication stress in association with genomic destabilisation, leading to clonal evolution of cells with abrogated defence systems^2^. The results presented here are consistent with these observations and demonstrate that polyphenols can mediate genome stability maintenance and cancer suppression.

Because genomic instability is inevitably induced in most cancers, our results imply that many cancers are theoretically preventable through genome stability maintenance, and that polyphenol consumption is a promising preventative option. In this study, the cancer-suppressive effects of polyphenols were evident in MMR-deficient mice. Although the positive effect of polyphenols on longevity observed in this study was significant, it was still limited. To suppress cancer development and efficiently extend the healthy lifespan, it will be important to clarify the mechanisms via which genome stability is maintained.

Resveratrol directly and indirectly activates a number of factors, including SIRT1, AMPK and ATM, with multiple consequences, including positive effects on metabolism^18^. In addition, the results presented here demonstrate that reduction of the DSB level and maintenance of genome stability, associated with the transient stabilisation of H2AX, are additional effects of resveratrol. Although the mechanism by which the DSB level is reduced by resveratrol is still unclear, it appears to differ from the general repair-induction process. In the current study, DSB reduction by resveratrol was not very efficient; although resveratrol reduced the DSB level, it did not effectively lead to complete repair. In addition, the speed of the DSB reduction by resveratrol was slower than that of the general repair-induction process and the repair of DSBs caused by γ-rays (2–5 Gy), which usually takes a few hours^12,27^, DSBs were typically not repaired 3 hr after the resveratrol treatment. Reduction of the DSB level by resveratrol might involve chromatin remodelling. In fact, we identified a transient stabilisation of H2AX, which is mediated by the chromatin remodelling factors SNF2H and SIRT6^12^, in resveratrol-treated MEFs. H2AX stabilization occurred between 1.5 and 6 hr after resveratrol treatment and was reduced by 24 hr post-treatment, corresponding to the time when the number of γH2AX foci was reduced.

In human, the peak serum/plasma concentrations of total resveratrol and its derivatives after ingestion of a single serving of red wine are typically lower than 1.5 μM^25^. Although multiple effects of resveratrol have been reported *in vitro*, many, including its pro-apoptotic effect on cancer cells, were seen at concentrations much higher than those typically seen in serum^43,44^. The concentrations of resveratrol used in the current study (1.25–2.5 μM) might be unphysiologically high, but are much closer to those seen in serum/plasma than the concentrations used in previous studies.

In summary, the results presented here suggest that regular consumption of resveratrol-associated polyphenols suppresses the incidence of cancers that arise with MSI by supporting DSB reduction and promoting genome stability.

## Methods

### Cell culture

MEFs were prepared from wild-type mice as described previously and were cultured using a Std-3T3 passage protocol^45^ or a modified protocol (Resv-3T3, Chlo-3T3 or MelRes-3T3), as described in the main text. To obtain immortalised cells, MEFs that reached the growth-arrested state (P8) were maintained without passaging, with a medium change every 3 days, until they exhibited immortal growth (IP1). All cells were cultured in Dulbecco’s Modified Eagle’s Medium (Nakarai) supplemented with 10% (v/v) foetal calf serum (Gibco).

### Cell treatments and analyses

DSBs that arose spontaneously during growth of cells under the Std-3T3 protocol were monitored. Resveratrol (Sigma), chlorogenic acid (Sigma), gnetin C (Yamada Bee Company Inc.), gnemonoside A (Yamada Bee Company Inc.) and gnemonoside D (Yamada Bee Company Inc.) were used for cell treatments as indicated. Melinjo resveratrol (Yamada Bee Company Inc.), a powdered extract of melinjo seed (Lot.YMP-M-160121) standardised to contain a minimum of 20% resveratrol derivatives, was also used; the extract included 0.1% *trans*-resveratrol, 2.5% gnetin C, 19.6% gnemonoside A, 4.3% gnemonoside D and 9.0% dextrin. Western blotting was performed as described previously^26^ and proteins were transferred to PVDF membranes for 2 hr. Immunofluorescence analyses were performed as described previously^26,27^ using a confocal laser microscope (Olympus FV10i). *P*-values were used to determine the statistical significance of differences between groups. The *P*-values shown in dot plots and bar graphs were calculated via two-tailed Welch’s *t*-tests.

### Antibodies

Antibodies against the following proteins were obtained from the indicated suppliers: β-actin (AC-74, Sigma), H2AX (A300-082A-1 for immunofluorescence and A300-082A-2 for immunoblotting, Bethyl Laboratories), H3 (MABI0301, MBL), γH2AX (JBW301, Upstate Biotechnology; 9718, Cell Signaling Technology), PCNA (ab29, Abcam), p-RPA32 (phosphorylated at Ser 33) (NB100-544, Noxus Biologicals), 53BP1 (PC712, Merck), α-tubulin (T6074, Sigma-Aldrich) and cleaved caspase-3 (9661, Cell Signaling Technology).

### Animal experiments

*Msh2*^−/−^ mice were fed the NMF non-purified diet (Oriental Yeast Co.) with or without 0.03% or 0.3% melinjo resveratrol (Yamada Bee Company Inc.). The mice were housed in plastic cages at a controlled temperature (23 °C ± 3 °C) and humidity (50% ± 10%) with a 12 hr:12 hr light:dark cycle. In the intestinal polyp formation experiment, the mice were given drinking water containing 0.2% K_2_BrO_3_. All animal experiments were approved by the Animal Research and Animal Care Committee of Tohoku University (2014AgA-059, 2016AgA-022-1). The experiments were conducted under the guidelines issued by this committee, in accordance with Japanese governmental legislation (2005) that establishes rules for the care and use of animals in animal studies. *P*-values were used to determine the statistical significance of differences between groups. The Kaplan-Meir curve data were analysed by a Log-Rank test followed by a Holm-Sidak test. The tumour number was analysed via a Student’s *t*-test.

## Supplementary information

Supplementary Information.

Supplementary Information2.
