# Supplementary material for: Resveratrol and its Related Polyphenols Contribute to the Maintenance of Genome Stability
Source: Sci Rep. 2020 Mar 25;10:5388. doi: 10.1038/s41598-020-62292-5 (PMC7096489; doi:10.1038/s41598-020-62292-5)
Supplement: Supplementary file 1 — Supplementary Information. [file 41598_2020_62292_MOESM1_ESM.docx]

**Supplementary information for**

**Resveratrol and its Related Polyphenols Contribute to the Maintenance of Genome Stability**

**Yusuke Matsuno, Yuko Atsumi, Md Alauddin, Md Masud Rana, Haruka Fujimori, Mai Hyodo, Atsuhiro Shimizu, Tomoki Ikuta, Hiroko Tani, Hidetaka Torigoe, Yoshimichi Nakatsu, Teruhisa Tsuzuki, Michio Komai, Hitoshi Shirakawa, Ken-ichi Yoshioka**

**This file includes Supplementary Figures 1–3.**


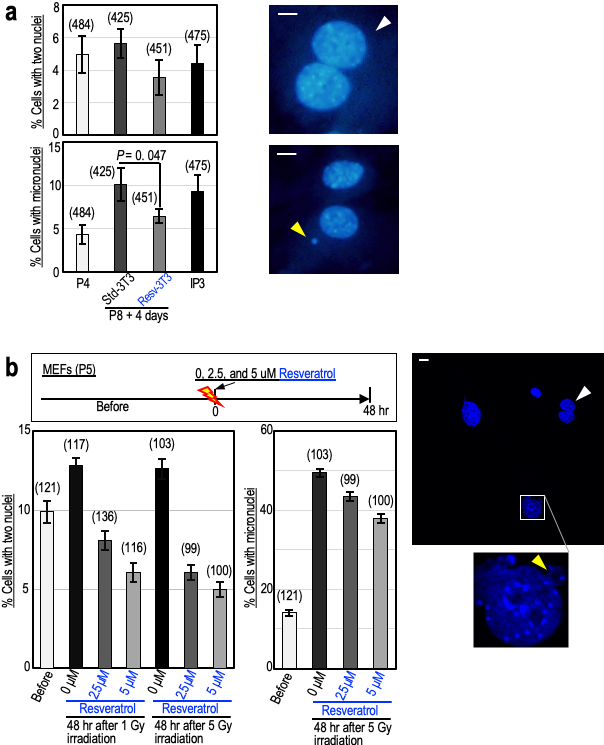


**Supplementary Figure S1. Resveratrol contributes to genome stability maintenance.** (a) Suppression of aberrant nuclei formation under the Resv-3T3 protocol (2.5 μM resveratrol). MEFs were cultivated as described in Figure 1a and the percentages of cells displaying two nuclei or micronuclei were determined. (b) The effects of resveratrol on genome stability maintenance after γ-ray irradiation. MEFs were treated as shown in the upper box. Nuclei were stained with DAPI (n numbers are indicated in the graph). Data in the graphs are represented as the mean ± s.d. Scale bars, 10 μm. A two-sample test for equality of proportions with continuity correction was used for the statistical analysis.


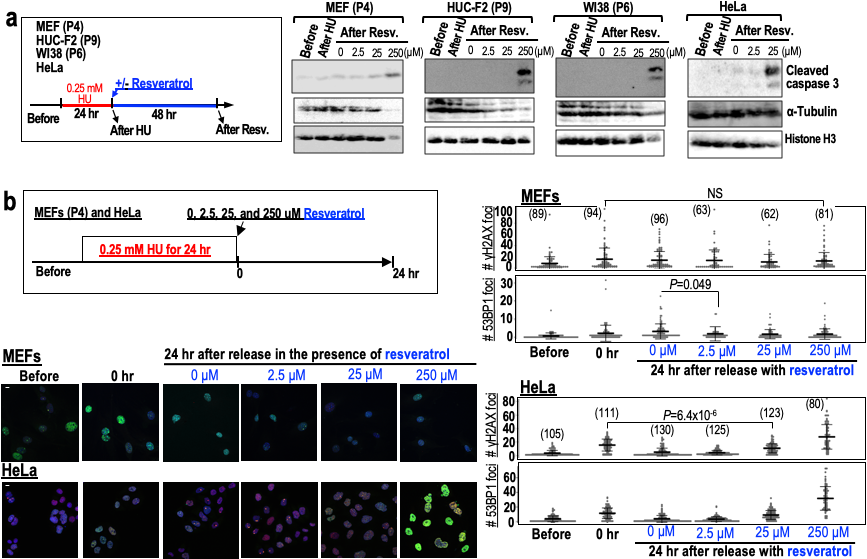


**Supplementary Figure S2. High concentrations of resveratrol induce apoptosis in various cell types.** (a) The induction of cleaved caspase-3 by high doses of resveratrol. The cells were treated with or without 0.25 mM hydroxyurea (HU) and with or without the indicated concentrations of resveratrol, as shown in the box on the left. Histone H3 and α-tubulin were used as controls. (b) The γH2AX foci statuses of resveratrol-treated cells. MEFs and HeLa cells were treated as shown in the upper box. The numbers of γH2AX foci detected by immunofluorescence are shown in the scatter plots (n numbers are indicated in the graphs). Data in the graphs are represented as the mean ± s.d. Scale bars, 10 μm. *P*-values were calculated by two-tailed Welch’s *t*-tests.


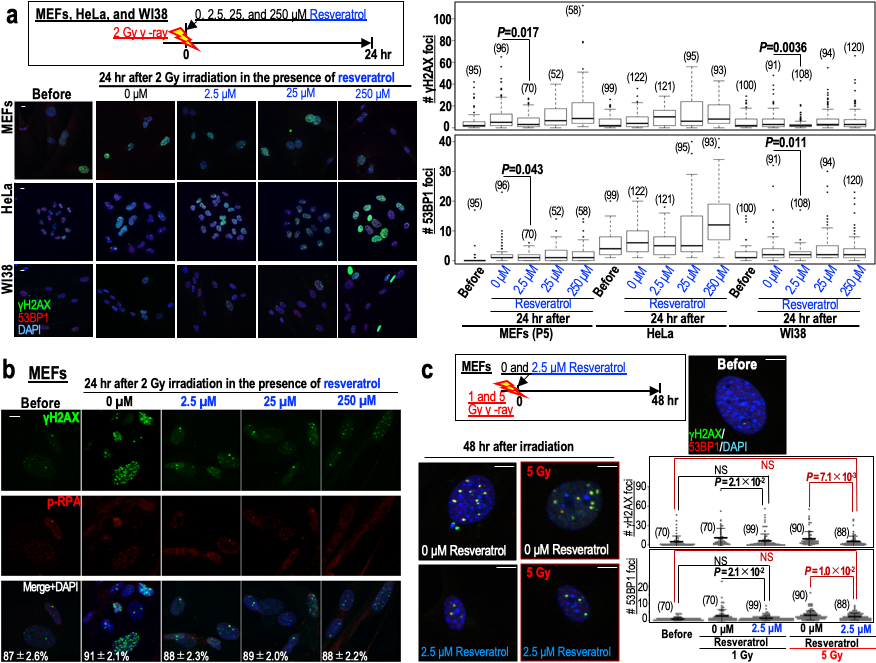


**Supplementary Figure S3. Low doses of resveratrol contribute to DSB reduction.** (a, b) The effects of resveratrol on the reduction of DSBs induced by 2 Gy γ-ray irradiation of MEFs (a,b), HeLa cells (a) and WI38 cells (a). The cells were treated as shown in the upper box. γH2AX, 53BP1 and p-RPA were detected by immunofluorescence (n numbers are indicated in the graphs). In (b), the percentages of the γH2AX foci that merged with p-RPA foci (mean ± s.e.) are indicated in each image. Scale bars, 10 μm. In (a), the box plots show the median, third and first quartiles, whiskers (median ± 1.5 times the interquartile range) and outliers. *P*-values were calculated by two-tailed Welch’s *t*-tests. (c) The effects of resveratrol on the reduction of DSBs induced by 1 or 5 Gy γ-ray irradiation of MEFs. The cells were treated as shown in the upper box. 53BP1 and γH2AX were detected by immunofluorescence (n numbers are indicated in the graphs). Data in the graphs are represented as the mean ± s.d. Scale bars, 10 μm. *P*-values were calculated by two-tailed Welch’s *t*-tests.
