# Supplementary material for: Resveratrol and its Related Polyphenols Contribute to the Maintenance of Genome Stability
Source: Sci Rep. 2020 Mar 25;10:5388. doi: 10.1038/s41598-020-62292-5 (PMC7096489; doi:10.1038/s41598-020-62292-5)
Supplement: Supplementary file 2 — Supplementary Information2. [file 41598_2020_62292_MOESM2_ESM.pptx]

## Slide 1
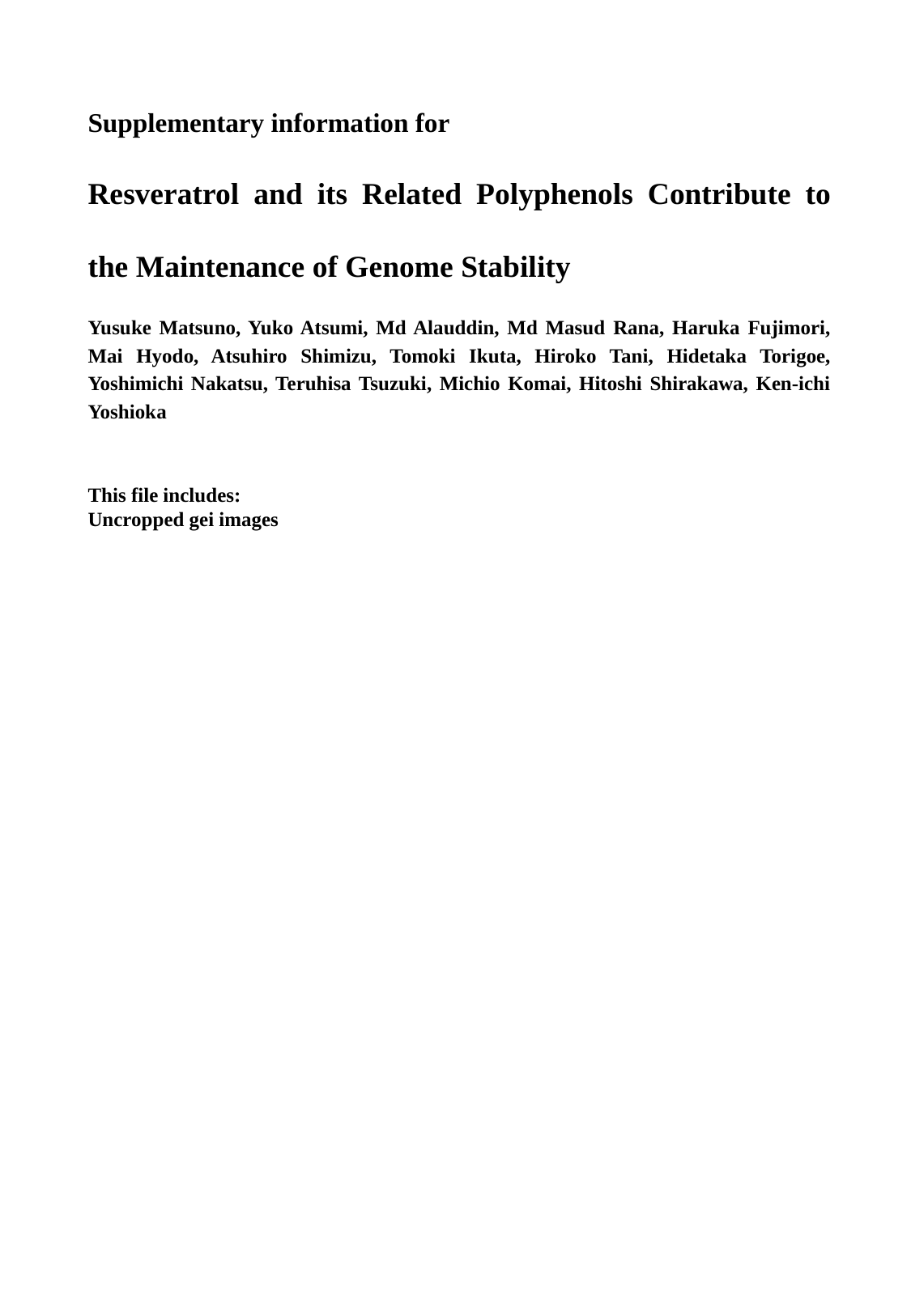

Supplementary information for
Resveratrol and its Related Polyphenols Contribute to the Maintenance of Genome Stability
Yusuke Matsuno, Yuko Atsumi, Md Alauddin, Md Masud Rana, Haruka Fujimori, Mai Hyodo, Atsuhiro Shimizu, Tomoki Ikuta, Hiroko Tani, Hidetaka Torigoe, Yoshimichi Nakatsu, Teruhisa Tsuzuki, Michio Komai, Hitoshi Shirakawa, Ken-ichi Yoshioka
This file includes:
Uncropped gei images

## Slide 2
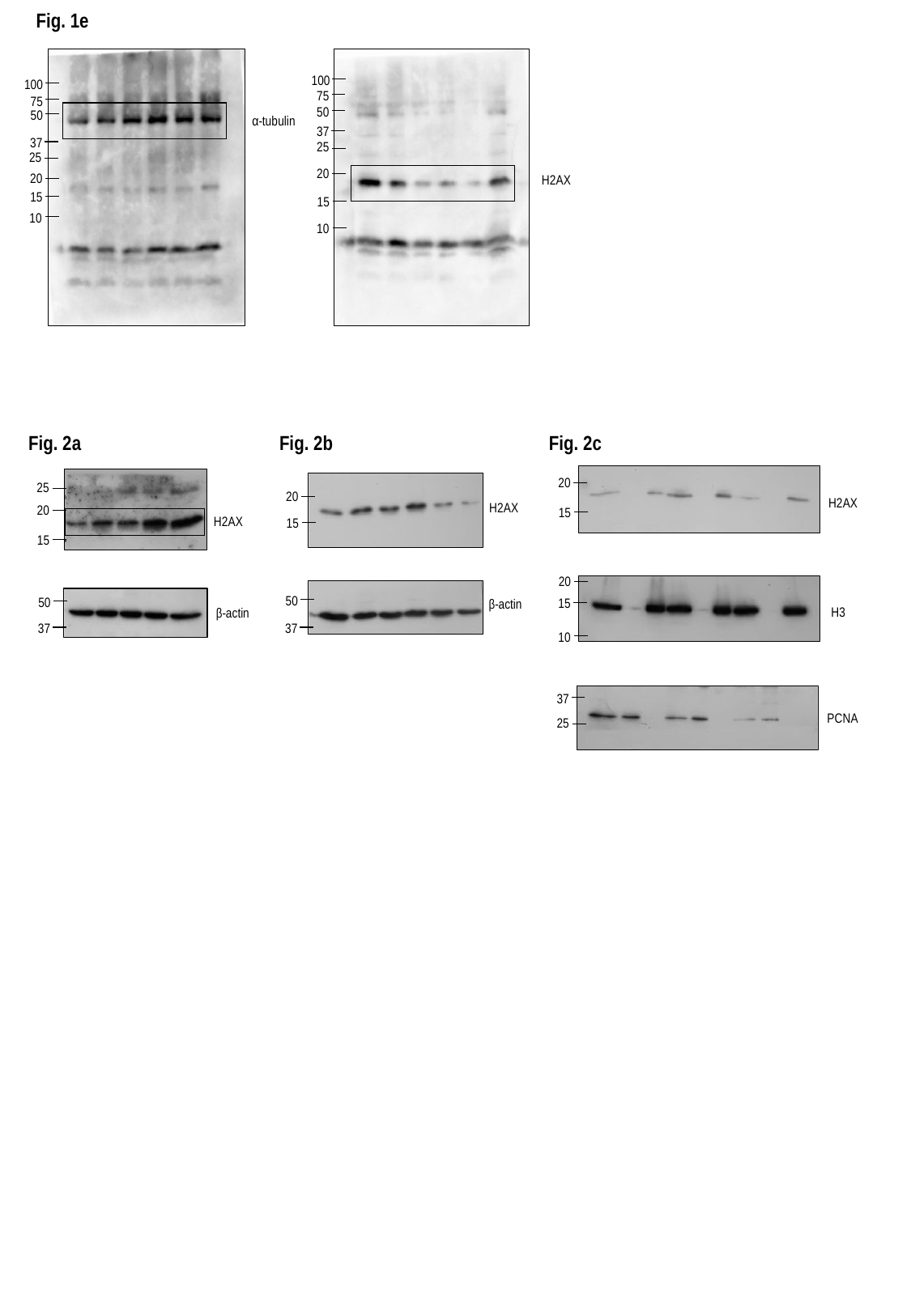

Fig. 1e
100
100
75
75
50
50
α-tubulin
37
37
25
25
20
20
H2AX
15
15
10
10
Fig. 2a
Fig. 2b
Fig. 2c
20
25
20
H2AX
H2AX
20
15
H2AX
15
15
20
50
50
15
β-actin
H3
β-actin
37
37
10
37
PCNA
25

## Slide 3
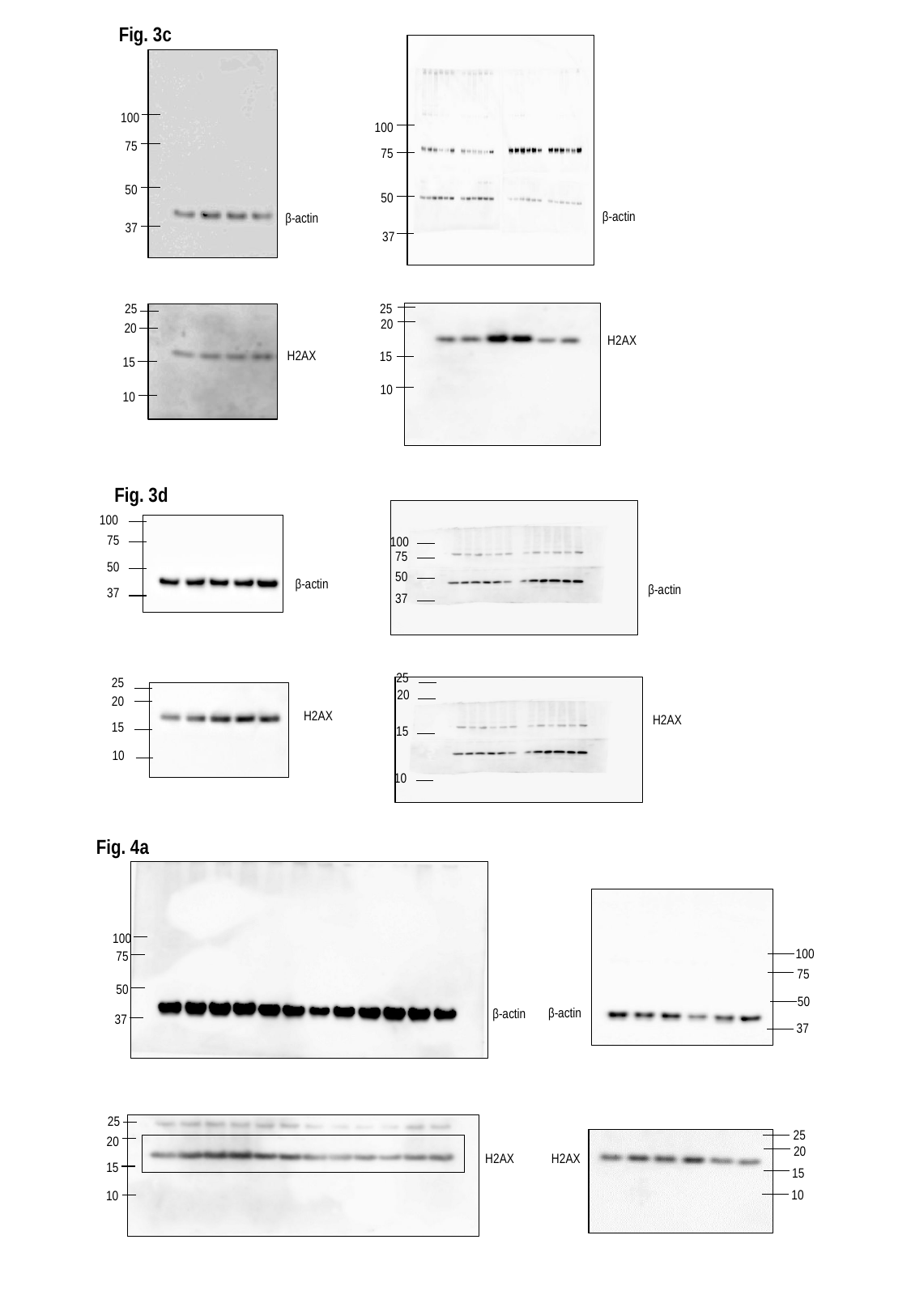

Fig. 3c
100
100
75
75
50
50
β-actin
β-actin
37
37
25
25
20
20
H2AX
H2AX
15
15
10
10
Fig. 3d
100
75
100
75
50
50
β-actin
β-actin
37
37
25
25
20
20
H2AX
H2AX
15
15
10
10
Fig. 4a
100
100
75
75
50
50
β-actin
β-actin
37
37
25
25
20
20
H2AX
H2AX
15
15
10
10

## Slide 4
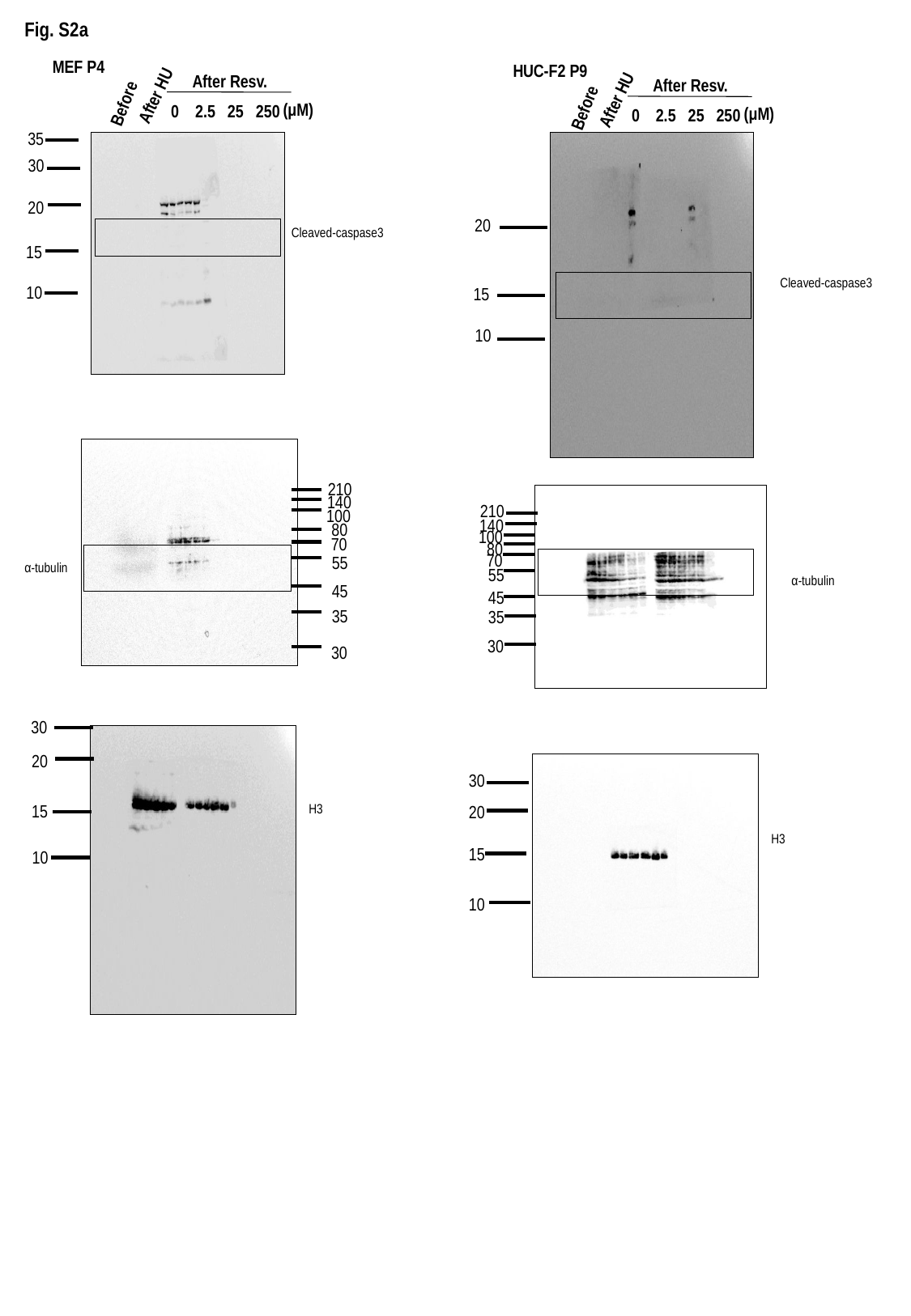

Fig. S2a
After Resv.
After HU
Before
(μM)
0 2.5 25 250
After Resv.
After HU
Before
(μM)
0 2.5 25 250
MEF P4
HUC-F2 P9
35
30
20
15
10
20
15
10
Cleaved-caspase3
Cleaved-caspase3
210
140
100
80
70
55
45
35
30
210
140
100
80
70
55
45
35
30
α-tubulin
α-tubulin
30
20
15
10
30
20
15
10
H3
H3

## Slide 5
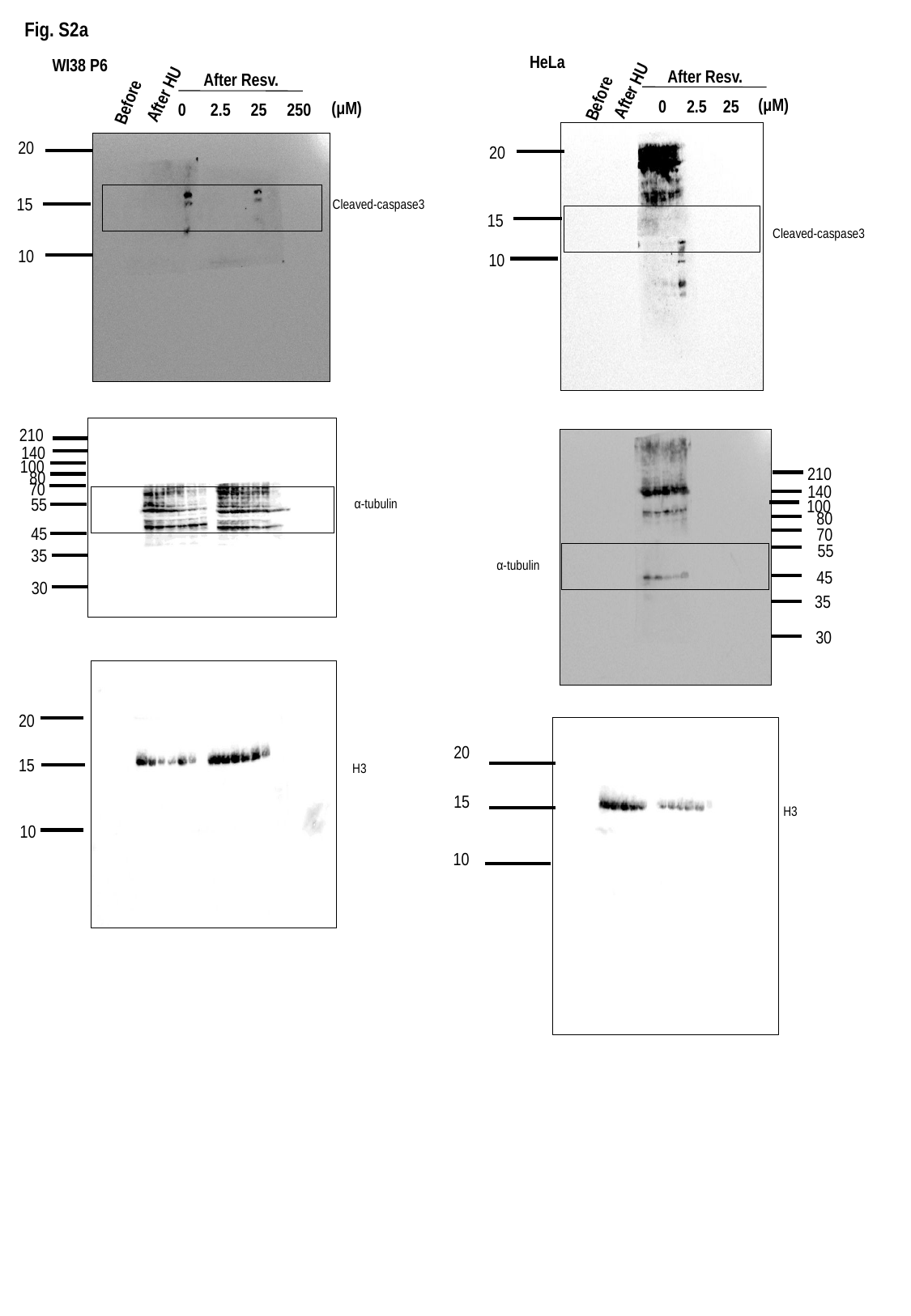

Fig. S2a
After Resv.
After HU
Before
(μM)
 0 2.5 25
After Resv.
After HU
Before
(μM)
0 2.5 25 250
HeLa
WI38 P6
20
15
10
20
15
10
Cleaved-caspase3
Cleaved-caspase3
210
140
100
80
70
55
45
35
30
210
140
100
70
55
45
35
30
80
α-tubulin
α-tubulin
20
15
10
20
15
10
H3
H3
